# Trends in vaccine investment in middle income countries

**DOI:** 10.1080/21645515.2019.1589287

**Published:** 2019-04-12

**Authors:** K. Onishchenko, S. Hill, M. Wasserman, C. Jones, M. Moffatt, L. Ruff, S. J. Pugh

**Affiliations:** a Health Economics and Outcomes Research, Consulting at McCann Health, London, UK; b Pfizer Inc., New York, NY, USA; c Health Economics and Outcomes Research, Consulting at McCann Health, Glasgow, UK; d Health Economics and Outcomes Research, Consulting at McCann Health, Macclesfield, Cheshire, UK; e Pfizer Inc., Collegeville, PA, USA

**Keywords:** Vaccination, immunization, expenditure, emerging, investment, coverage

## Abstract

Although a proven and effective preventive health measure, childhood immunization programs remain vulnerable to budgetary pressures. Sustainable financing of immunization programs is an important issue that presents a challenge for middle-income countries (MIC) in particular, in part due to technological advances meaning more vaccines are available. This study aimed to analyse trends in immunization program investment across 15 MIC selected based on availability of data, income level classification, and regional representativeness. We assessed investment trends in relation to vaccine coverage, vaccine access, and broader health indicators. Immunization and expenditure data were obtained from the World Health Organisation (WHO) database and the WHO UNICEF Joint Reporting Form and WHO Vaccine Product, Price and Procurement from 2006–2016. We calculated a weighted average index of vaccine commitment (WAIVC) based on vaccine coverage, vaccine scope, and weighted by vaccine innovation measured by approximating vaccine expenditure. Correlation analyses were conducted between immunization expenditure per-capita and each WAIVC, infant mortality and life expectancy. Correlation analyses at a global and individual country level indicate an improvement in immunization access, vaccination commitment measured by WAIVC, and scope of available vaccines in countries with sustained increases in vaccination funding. Increases in national immunization expenditure were correlated with reduced infant mortality and increased life expectancy. Vaccine expenditure comprises a small proportion (less than 2%) of total healthcare spending and has not uniformly increased in accordance with the scope of available vaccines. The present analysis supports the premise that countries with consistent increases in vaccine expenditure have increased vaccine coverage and commitment measured by WAIVC and improved broader health outcomes, indicating the value of sustained investment in vaccination for improved population health. The benefits of vaccine expenditure in this holistic fashion are critical to inform policy decisions on national budget allocation for vaccine funding.

## Background

Vaccines are a leading public health achievement of our time and remain one of the most effective preventive health measures to date. The benefits of vaccination continue to be recognized; as of 2015, the United Nations (UN) identified access to affordable essential vaccines as a significant target for improving health outcomes by 2030.^1^ Vaccinated populations also achieve broader benefits such as greater educational attainment, increased productivity,^2^ and ultimately economic growth and increased per capita gross domestic product (GDP).^3^ Despite these broad benefits, vaccination programs typically account for only a small proportion of national health expenditure, yet continue to be vulnerable to budgetary pressures.^4^ The continued investment in vaccines is imperative to achieving international goals of reducing vaccine-preventable morbidity and mortality.^5^

Sustained investment in immunization programs is particularly pertinent for emerging economies, where vaccine-preventable diseases continue to be a leading cause of morbidity and mortality.^6^ However, due to financial constraints, low-to-middle income countries (LMIC) and middle-income countries (MIC) are often less likely to include new vaccines as part of their immunization programs.^7^ Gaps in funding and coverage are historically evident between older and newer-generation vaccines due to differences in cost and vaccine acceptance, which is higher for newer than older-generation vaccines. These differences in funding and coverage are typically greater within lower income than in higher income countries.^8–12^ Over time, a lack of financing for vaccines may result in sub-optimal immunization policies without coverage for life-saving vaccines. This may be exacerbated in middle-income economies, which predominantly finance vaccines from national budgets. In contrast, many low-income countries often receive funding for some vaccines from non-governmental organizations such as Gavi, a public-private global health partnership committed to increasing access to immunisation in poor countries.^8^

In MIC not eligible for Gavi funding, defined as a gross national income (GNI) per capita between $1,006 to $12,235, uncertainty in financing and the absence of efficient channels through which investment is delivered may inhibit the introduction of new vaccines; though, the exact reasons have yet to be fully explored.^7^ Low uptake of newer-generation vaccines can be in part attributed to relatively high purchasing costs and challenges around planning to support National Immunisation Programs (NIPs) for new vaccines such as the potential logistical complexities associated with introducing a new vaccine (including infrastructure, budgetary and human resource planning).^7,9,10^ Yet, the undervaluation of newer-generation vaccines and the unrecognized broad benefits achieved from a NIP may also contribute to the relatively slow uptake in developing countries.

Maintaining coverage for existing vaccines and introducing new vaccines in developed countries has been shown to be greatly leveraged by the level of investment in NIPs.^11^ The relationship between national health budgets and their impact on immunization programs in high-income countries has been well documented. The findings from a recent study examining vaccine investment and coverage in Western Europe reported a net trend towards a decrease in national vaccine spending,^11^ despite the relatively low investment per individual.^4^ While this evidence has been limited to developed economies, the question may be of particular importance to middle income economies as they continue to face budgetary constraints^2^ and are either ineligible for or transitioning from funding provided by the Gavi Alliance. As countries will be required to support their immunization programs without international aid,^2^ it is important to understand the current trends in vaccine investment.

This study aimed, firstly, to describe trends over time in national immunization expenditure and vaccine utilisation as well as the relationship between expenditure and overall vaccine commitment in middle-income countries. Our secondary objective was to describe the ecological relationship between vaccine investments and broader global health outcomes, such as infant mortality and life expectancy, goals designated by the UN Sustainable Development Initiative.^1^

## Results

As of 2006, across vaccines recommended for inclusion in all country NIPs,^13^ vaccine coverage was highest for established vaccines such as DTP (72–99% coverage) and BCG (88–99% coverage) and lowest for newer-generation vaccines such as PCV, IPV, Hib and rotavirus vaccines (0%-99% coverage) (Table 1). As of 2016, vaccine coverage in general increased and new vaccines were added to the NIPs in the majority of countries. However, newer-generation vaccines continued to demonstrate lower and less consistent coverage into 2016 (Supplementary Table 2).

**Table 1.** Summary of markets included in the current analysis.

Country	Geographic Region	Income Status	Vaccine Financing
**Indonesia**	Asia-Pacific	Low-Middle Income	Government finance increasing (89% in 2016) – in transition from Gavi
**Malaysia**	Asia-Pacific	Upper-Middle Income	Lack of recent data (80% financed by government in 2011)
**Philippines**	Asia-Pacific	Low-Middle Income	100% financed by government
**China**	Asia-Pacific	Upper-Middle Income	100% financing by government
**Thailand**	Asia-Pacific	Upper-Middle Income	100% financed by government
**Vietnam**	Asia-Pacific	Low-Middle Income	Approximately 50% financed by government in 2016 – in transition from Gavi
**Sri Lanka**	Asia-Pacific	Low-Middle Income	State funding increased from 74% to 100% in 2016 (following Gavi transition)
**Kazakhstan**	Europe	Upper-Middle Income	100% financed by government
**Brazil**	Latin America	Upper-Middle Income	Generally 100% financed by government (fell to 88% in 2016)
**Colombia**	Latin America	Upper-Middle Income	100% financed by government
**Egypt**	Africa and Middle-East	Low-Middle Income	100% financed by government
**Morocco**	Africa and Middle-East	Low-Middle Income	100% financed by government
**Jordan**	Africa and Middle East	Low-Middle Income	100% financed by government
**Bulgaria**	Europe	Upper-Middle Income	100% financed by government
**Romania**	Europe	Upper-Middle Income	100% financed by government

**Table 2.** Reported vaccine coverage rates in 2006 and 2016, according to the WHO/UNICEF immunization joint reporting form.

		BCG (%)	DTP3 (%)	HepB3 (%)	Hib3 (%)	IPV1 (%)	MCV2 (%)	PCV3 (%)	Pol3 (%)	RCV1 (%)	RotaC (%)	YFV (%)	JE (%)
**Indonesia**	**2006**	88	72	66	0	0	50	0	78	0	0	0	0
	**2016**	81	79	79	79	2	56	0	80	0	0	0	0
**Malaysia**	**2006**	98	95	95	89	0	90	0	95	95	0	0	0
	**2016**	98	98	98	98	91	99	0	98	96	0	0	98
**Philippines**	**2006**	91	88	77	0	0	0	0	88	0	0	0	0
	**2016**	76	86	86	86	37	66	36	72	80	0	0	0
**China**	**2006**	92	93	91	0	0	94	0	94	0	0	0	0
	**2016**	99	99	99	0	0	99	0	99	99	0	0	99
**Thailand**	**2006**	99	99	96	0	0	94	0	99	99	0	0	87
	**2016**	99	99	99	0	0	95	0	98	96	0	0	92
**Vietnam**	**2006**	95	94	93	0	0	0	0	94	0	0	0	95
	**2016**	95	96	96	96	0	95	0	95	99	0	0	95
**Sri Lanka**	**2006**	99	98	98	0	0	98	0	98	98	0	0	70
	**2016**	99	99	99	99	99	99	0	99	99	0	0	99
**Kazakhstan**	**2006**	99	99	99	0	0	99	0	99	99	0	0	0
	**2016**	95	82	82	82	93	99	97	82	99	0	0	0
**Brazil**	**2006**	99	99	99	97	0	55	0	99	99	47	34	0
	**2016**	99	86	86	86	80	72	94	98	96	94	39	0
**Colombia**	**2006**	96	93	93	93	0	85	0	94	95	0	88	0
	**2016**	88	91	91	91	92	87	89	91	93	90	92	0
**Egypt**	**2006**	99	98	98	0	0	97	0	98	97	0	0	0
	**2016**	96	95	95	94	0	96	0	95	95	0	0	0
**Morocco**	**2006**	95	97	95	0	0	0	0	97	92	0	0	0
	**2016**	99	99	99	99	95	99	98	99	99	99	0	0
**Bulgaria**	**2006**	98	95	96	0	0	93	0	96	96	0	0	0
	**2016**	96	92	91	92	0	88	90	92	92	0	0	0
**Romania**	**2006**	99	97	99	0	0	96	0	97	95	0	0	0
	**2016**	84	89	90	89	0	76	0	89	86	0	0	0
**Jordan**	**2006**	95	98	98	98	0	88	0	98	88	0	0	0
	**2016**	99	98	98	98	99	99	0	98	99	97	0	0

Government expenditure on vaccines was highly variable across countries. On average, there was a trend towards increasing per capita expenditure from 2008 to 2012, where the largest increases occurred in Malaysia, Bulgaria, and Kazakhstan. Then, in years 2012 to 2014, there was a trend towards a stagnant or decreasing per capita expenditure on vaccination (Supplementary Figure 1).

**Figure 1.**
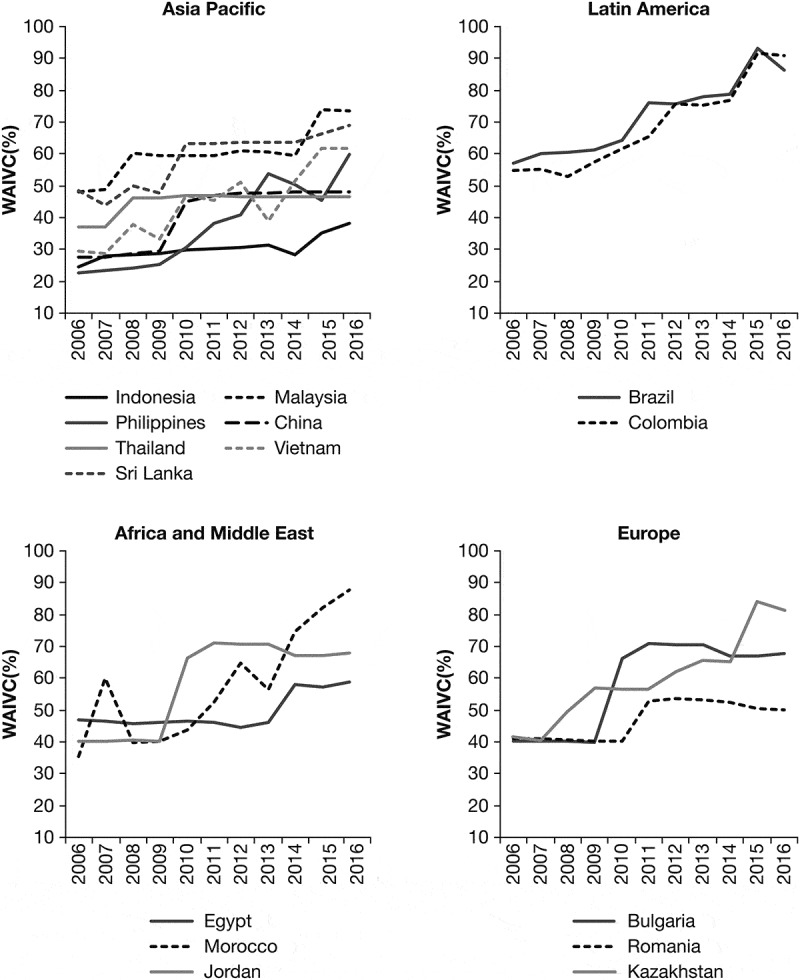
Calculated Weighted Average Index of Vaccine Commitment (WAIVC) from 2006–2016.

Figure 1 depicts country-specific trends in vaccine commitment; defined by the calculated weighted average index of vaccine commitment, which accounts for government expenditure on vaccines as well as the number of vaccines and uptake of vaccines included in immunisation programs over time. Nearly all investigated countries had an increasing trend for vaccine commitment from 2006 to 2016. Variability existed between countries in the magnitude of vaccine commitment improvement over time, however most countries had introduced at least one new vaccine to their immunization programs since 2006. For example, Vietnam has demonstrated the most significant improvement of its vaccination commitment index from 29% in 2006 to 62% in 2016. In contrast, some countries had stagnant vaccine commitment from 2008–2016, with the weighted average index of 46% and 46.5% in 2008 and 2016 respectively.

**Figure 2.**
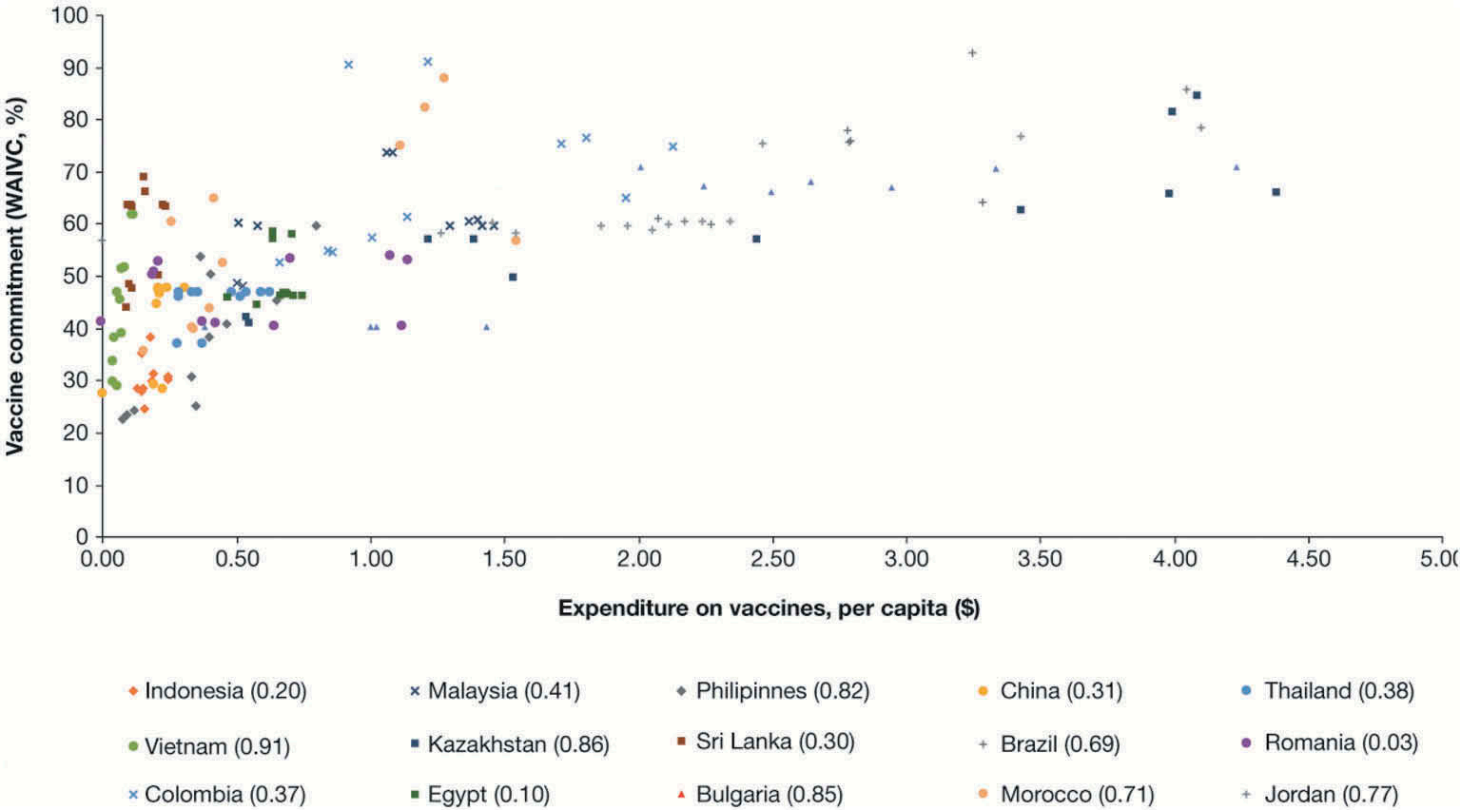
The evolution of vaccine commitment in relation to per capita government expenditure on vaccines in 2006–2016. *Individual correlations between vaccine commitment and per capita government expenditures on vaccines are shown in square brackets

The relationship between the index of vaccine commitment and per-capita government expenditure was also investigated (Figure 2). Correlation analyses indicated a general improvement in immunization scope and vaccination commitment (WAIVC) with sustained increases in vaccination funding. Specifically, vaccine commitment was found to be positively associated with per capita government expenditure in all 15 emerging economies, with a positive overall correlation coefficient of 0.52 between vaccine commitment and per capita government expenditures (Supplementary Table 3)^17,29,30^. The average estimated correlation coefficient values were consistent across regions, with Asia, Latin America, Africa and the Middle East, and Europe demonstrating similar correlations within 0.4–0.6 range. However, the estimated correlation coefficient between vaccine commitment index and per capita government expenditure varied across individual countries (Supplementary Table 3). The majority of countries, including the Philippines, Vietnam, Kazakhstan, Brazil, Morocco, Bulgaria and Jordan, demonstrated a strong correlation (index exceeding the 0.602 critical value at a 0.05 level of significance for a two-tailed test of Pearson’s correlation), while fewer countries showed a moderate (0.521–0.602) or low correlation (less than 0.521)^1^,^14^ Scenario analyses assessing the correlation between vaccine commitment and total government expenditure also followed a similar dynamic to per capita expenditure, demonstrating positive association between expenditure and coverage.

In correlation analyses between per capita expenditure and broader health outcomes, results trended in the hypothesized direction (Supplementary Table 3). The majority of the estimated correlation coefficients between per capita government expenditures and infant mortality showed strong negative association. All the estimated correlation coefficients showed positive association between life expectancy and per capita government expenditures, with most demonstrating a high correlation coefficient >0.6.

## Discussion

Across the 15 investigated countries from Asia, Latin America, Europe, Africa, and the Middle East, an increasing investment in vaccination was positively correlated with a higher level of vaccine commitment. Increasing vaccine commitment indicates a country’s ability to sustain vaccine uptake under the current vaccination schedule while continuing to introduce newer-generation vaccines into the immunization programs. Furthermore, results indicate a broader public health value of sustained vaccine investment as countries with greater vaccine commitment had lower infant mortality and better life expectancy.

Despite a general trend towards improvement in vaccine commitment between 2006 to 2016, individual correlations between per capita expenditure and vaccine commitment varied by country. Various market fundamentals have the potential to influence this relationship. For example, fluctuations in GDP levels may impact the sustainability of health care sector financing for NIPs in a country.^15^ Also, increasing pressure from a growing anti-vaccination movement is a challenge driving reduced immunization rates in several countries.^16^ Another potential explanation may be due to the required health care structural reforms and facility upgrades required to implement new NIPs, which may delay observed relations between investment and uptake.^17,18^ The remaining coverage gaps between older and newer-generation vaccines are typically greater within low to middle income countries, but still exist in higher income economies.^13,19^ In contrast, developing countries such as Vietnam have consistently maintained a Hib coverage at a level of over 90%, largely due to Gavi disbursements.^2^,^13,20^

Morocco, Kazakhstan and Vietnam showed the greatest correlations between expenditure, index of vaccine commitment and health outcomes. Morocco and Kazakhstan demonstrated over a 50% improvement in vaccine commitment from 2006–2016, after introducing Hib and PCV vaccines, while Vietnam vaccine commitment increased by 120% due in part to a sustained increase in government expenditure on vaccines and Gavi support demonstrating a remarkable success of the implementation of the NIPs. These positive market dynamics observed in Morocco, Kazakhstan and Vietnam suggest that consistently greater spending on vaccines is associated with a more successful national vaccination program.

This publication is among the first to examine the correlation between a vaccine commitment index, expenditure and other outcomes such as infant mortality and life expectancy in emerging economies. Our results on the trends in investment and vaccine coverage are generally consistent with a recent study in Europe, which concluded that allocation towards vaccine budgets has not sustained increases in the total health care budget.^11^ However, one key difference between this and the current analysis was the use of a commitment index designed to capture a country’s efforts towards inclusion of new vaccines and sustained vaccine coverage in the latter. Our index elaborated on the individual vaccine coverage trends by demonstrating an overall commitment across emerging economies towards increasing vaccine access. Our findings indicating a broader public health value of vaccine commitment are supported by previous studies reporting decreased infant mortality after PCV13, influenza, meningococcal, and rotavirus vaccines;^21–24^ however, the relationship between individual vaccination policies and life expectancy has been investigated to a lesser extent. In addition, the broader value of vaccine commitment was also demonstrated in a recent study suggesting an increase in GDP per dollar invested in vaccines, likely due in part to the impact of vaccines on disease prevention and infant survival, enabling a more productive work force.^16,25^ Taken together, these findings could be encouraging from a governmental perspective, through the implication of improved population health, survival, and productivity.^3,26^ While these findings do highlight the value of successfully developing and implementing the NIPs in emerging economies, it is important to note that vaccination is not the only method to improve broader health and socio-economic outcomes.

### Strengths and limitations

This study is strengthened by the inclusion of emerging global economies across a broad geographic and economic range. Our findings are based on existing databases, but may be limited by the self-reporting mechanisms through which the investigated countries report their data. The findings must be interpreted with caution as they are descriptive rather than causal. In addition, since we assessed countries within a GNI per capita range and used vaccine commitment data by country from open sources, we cannot account for within country income variation and how this may impact correlations. The WHO data set on total expenditure on vaccines was incomplete with data points missing for several countries. Therefore, considering existing data limitations we used government expenditure on vaccines as a better proxy for vaccine commitment at national level. The limitation of this approach; however, is that in countries with a larger middle-income class who have access to private sector vaccination, our findings may underestimate the true relationship between vaccine investment and access at least for some income groups as higher real uptake may be influencing these findings. We conducted a Pearson correlation, which is neither distributionally robust, nor multicollinearity and outlier resistant; however, it allows to quantify the strength of association between the variables of interest. The robustness of correlation analyses was increased through the use of a weighted average index of vaccination commitment. Through this approach, the dimensions describing vaccination performance and vaccination financing were combined to more accurately quantify the degree of vaccination commitment at a country level. The weighted index was applied in this study to account for the shares of spending on each vaccine in order to better approximate commitment to newer-generation vaccines, thus weighting the impact of each vaccine in the vaccination coverage domain and accounting for individual country variation. While there are limitations with this approach and may be technical arguments for weighting some dimensions and sub-dimensions more than others, constructing reasonable weights would require greater empirical evidence on the connection between each measure and the outcome of interest, which is currently unavailable.^27^

## Conclusion

Sustained increases in vaccination funding is a key prerequisite to potential improvements in immunization access, higher level of vaccination commitment, and wider scope of available vaccines in emerging economies. Yet, vaccine expenditure has not increased in all countries in accordance with the scope of available vaccines despite comprising only a small proportion (less than 2%) of total healthcare spending. Our analysis supports the premise that both consistent and inclusive immunisation programs for newer-generation vaccines can yield not only broad-based health benefits but also encourage the related societal value of saving lives in infancy and in preserving a national workforce capable of providing productivity returns. The benefits of vaccine expenditure in this holistic fashion are critical to inform policy decisions on national budget allocation for vaccine funding.

## Methods

The World Health Organisation (WHO) maintains global databases to monitor the impact of strategies for reducing morbidity and mortality of vaccine-preventable diseases in order to inform vaccination policy and programs. We abstracted data from two of these databases for the current analysis, WHO/UNICEF Immunization Joint Reporting Form (JRF) and The WHO Vaccine Product, Price and Procurement Database (V3P). The JRF captures data on the type and coverage of vaccines included in a country’s NIP, and is updated on a yearly basis based on self-reports from country-specific national immunization program staff. The V3P is a platform that provides un-biased data on vaccine product, price, and procurement and is based on country reported estimates. Data in the V3P includes estimates of average vaccine cost per income level and national immunization expenditure for vaccines supported through public funding.^12,13^ We used both of these databases to abstract country-specific vaccine uptake, coverage, and spending from 2006–2016.

We limited the current analysis to countries that met the following criteria: an emerging market or pre-emerging market,^3^ an income level classification between low to middle income and upper-middle income,^4^ had sufficient public data by year on vaccine uptake, coverage, and expenditure, and was representative of one of the following regions, Asia-Pacific, Europe, Africa, Middle-East, and Latin America. In the majority of selected countries, health services were wholly financed by the government; however, three countries (Indonesia, Vietnam and Sri Lanka) included in the analysis are transitioning from Gavi support and therefore, have additional sources of vaccine funding. The current analysis was limited to 15 selected countries that met these criteria (Table 2).

The vaccines included in each country’s NIP differ; therefore, to make direct comparisons of vaccine utilization equitably across all countries, we defined a benchmark for optimal vaccine utilization. Optimal vaccine utilization was based on the global WHO recommendations for immunization, accounting for region-specific recommendations (e.g. Japanese encephalitis vaccine and yellow fever vaccine).^28^ HPV and Meningococcal vaccines were excluded from analyses due to the lack of available coverage data from databases.

To describe country-specific vaccine uptake, we reported the uptake for each vaccine in 2006 and 2016 and calculated the change in uptake between these two time points (Supplementary Table 2). While vaccine uptake is one measure of a country’s vaccine utilisation, we aimed to define the continued commitment to vaccination through a broader index. We defined vaccine commitment as a measure of commitment towards national and global prevention, control, and where possible, elimination of vaccine-preventable diseases. To quantify vaccine commitment, we modified an existing index by Glassman et al.^27^ to capture 3 components over time: 1) the number of vaccines included in a vaccination program 2) vaccine uptake 3) innovation.

The number of vaccines included in a program was calculated out of a total of 10 core vaccines and 2 regional vaccines, consistent with the WHO recommendations for immunization. Some vaccines (e.g. human papilloma virus (HPV) vaccine) were excluded from the analysis due limited uptake data across all 15 identified countries. Vaccine uptake was calculated as the percent coverage of each vaccine. Innovation was estimated as the introduction of newer-generation vaccines into an NIP. Since newer-generation vaccines are often more expensive and comprise a larger portion of total immunization budgets, we approximated newer-generation vaccines based on the proportion of budget allocated to a specific vaccine, defined as >+1SD from the mean proportion of budget allocation. We used the V3P data to estimate the proportion of spending on each vaccine to calculate weights. However, data from the V3P on vaccine expenditure is de-identified by country,^12^ but does include an identifier for a country’s income status. Therefore, we calculated weights for the proportion of spending by overall income groups, lower- and upper-middle income, and applied the weights uniformly to countries within these income categories. We calculated four key sets of weights with the baseline index including yellow fever (Brazil and Colombia) and Japanese encephalitis vaccines (China, Malaysia, Thailand, Vietnam and Sri Lanka) in countries where these vaccines were recommended by the WHO (Supplementary Table 1).

According to these 3 components, the following index was calculated. The baseline index of vaccination commitment (IVC) for country (*I)* and vaccine (*D)* is defined below where $V_{i}^{s}$is vaccine uptake (*s)* in country (*i)* and $\max\left\{V_{s}\right\}$is the maximum uptake (100%). WHO evidence on vaccine pricing and procurement^12^ was used to weight the IVC index for the inclusion of a newer-generation vaccine in an immunization program, where *w_i_* is the average proportion of spending on vaccine *i* in total vaccine budget in a specified country income group.

$$WAIVC_{i}^{D}=\overset{s}{\underset{s=1}{\sum }}w_{i}\frac{V_{i}^{s}}{max\left\{V_{s}\right\}}$$

To describe the country-specific commitment to vaccination, we plotted the calculated WAIVC index over time to demonstrate the change in vaccine commitment from 2006–2016. We described the relationship between vaccine expenditure and vaccine commitment index by firstly, plotting these two variables for each country across the specified time horizon. Secondly, to quantify the relationship between observed trends, we supplemented the analysis with the calculations of Pearson’s correlation coefficient, a useful descriptor of the degree of linear association between the two variables. If the coefficient is near zero, there is no correlation, while a coefficient of −1 or +1 indicates a strong negative or positive relationship, respectively. The significance level was assessed against critical values for a two-tailed test of Pearson’s correlation for 11 pairs of variables for the investigated period of 2006–2016.^14^, ^5^ While the correlated variables both include a component of expenditure, they capture different attributes and are not inherently correlated as they reflect different level of aggregation at national and global levels. Specifically, the WAIVC index by construction is capturing the more aggregated level of vaccination commitment as a proportion, corrected for the distribution of vaccine spending across a pool of relevant market archetypes. Vaccine expenditure is a dollar amount and is based on total annual vaccine spend by country and therefore demonstrates the direct benefits of the society from country-specific vaccination policies.

Lastly, to assess the ecological relationship between vaccine expenditure and broader health outcomes, we conducted a series of correlation analyses at the national, regional and global levels with under 5 infant mortality and life expectancy obtained from the World Bank.^17,30^

## Data Availability

The datasets analysed during the current study are available in the World Health Organisation (WHO) Vaccine Product, Price and Procurement (V3P) Database (available at: http://www.who.int/immunization/programs_systems/procurement/v3p/platform/module1/en/) and WHO/UNICEF Estimates of National Immunization Coverage (WUENIC) (Available at: http://www.who.int/immunization/monitoring_surveillance/routine/coverage/en/index4.html)

## Notes

1. The correlation bounds were defined using the level of significance for two-tailed test for Pearson correlation. Specifically, we defined ‘moderate’ bound for 0.05–0.1 level of significance and ‘low’ bound for lower than 0.1 level of significance for two-tailed test for a given number of 11 pairs of variables and 9 degrees of freedom.

2. Gavi are specifically targeted at improving coverage of under-utilised vaccines in low-income economies. According to Gavi eligibility criteria countries are eligible to apply for Gavi support when their Gross National Income (GNI) per capita is below or equal to US$ 1,580 on average over the past three years (according to World Bank data published every year on 1 July). 47 countries will be eligible to apply for Gavi support in 2018 based on a Gross National Income (GNI).^32^

3. An emerging market is a market in the process of rapid growth and development with lower per capita incomes and less mature capital markets than developed countries. A pre-emerging or frontier market is a subset of the emerging market category. Specifically, a frontier market is one with little market liquidity, marginally developed capital markets, and lower per capita incomes vis à vis the more developed emerging markets like Brazil and China.^33^

4. For the 2018 fiscal year, low-income economies are defined as those with a gross national income (GNI) per capita, calculated using the World Bank Atlas method, of $1,005 or less in 2016; lower middle-income economies are those with a GNI per capita between $1,006 and $3,955; upper middle-income economies are those with a GNI per capita between $3,956 and $12,235; high-income economies are those with a GNI per capita of $12,236 or more. As of 1 July 2016, low-income economies are defined as those with a GNI per capita, calculated using the World Bank Atlas method, of $1,025 or less in 2015; lower middle-income economies are those with a GNI per capita between $1,026 and $4,035; upper middle-income economies are those with a GNI per capita between $4,036 and $12,475; high-income economies are those with a GNI per capita of $12,476 or more.^31^

5. The following critical values for the level of significance for two-tailed test for N = 11 number of pairs and N-2 = 9 degrees of freedom were used: 0.521 for 0.1 level, 0.602 for 0.05 level and 0.735 for 0.01 level.^14^
