# Supplementary material for: Trends in vaccine investment in middle income countries
Source: Hum Vaccin Immunother. 2019 Apr 12;15(10):2378–85. doi: 10.1080/21645515.2019.1589287 (PMC6816376; doi:10.1080/21645515.2019.1589287)
Supplement: Supplemental Material [file khvi-15-10-1589287-s001.docx]

# Supplementary Tables and Figures

Supplementary Table 1: Calculated weights for the Weighted Average Index of Vaccination Commitment (WAIVC)

| Vaccine | Baseline weight | | weights without JE and YFV | | weights without JE | | weights without YFV | | |  |
| --- | --- | --- | --- | --- | --- | --- | --- | --- | --- | --- |
|  | UMIC | LMIC | UMIC | LMIC | UMIC | LMIC | | UMIC | LMIC | |
| BCG | 0.011 | 0.022 | 0.013 | 0.024 | 0.012 | 0.024 | | 0.011 | 0.023 | |
| DTP3 | 0.095 | 0.101 | 0.110 | 0.109 | 0.106 | 0.107 | | 0.098 | 0.102 | |
| HepB3 | 0.081 | 0.086 | 0.093 | 0.094 | 0.090 | 0.092 | | 0.083 | 0.088 | |
| Hib3 | 0.136 | 0.127 | 0.156 | 0.139 | 0.151 | 0.136 | | 0.140 | 0.130 | |
| IPV1 | 0.133 | 0.054 | 0.153 | 0.059 | 0.148 | 0.058 | | 0.137 | 0.055 | |
| MCV2 | 0.079 | 0.097 | 0.091 | 0.105 | 0.088 | 0.103 | | 0.082 | 0.098 | |
| PCV3 | 0.146 | 0.203 | 0.168 | 0.220 | 0.162 | 0.216 | | 0.150 | 0.206 | |
| Pol3 | 0.022 | 0.037 | 0.025 | 0.040 | 0.024 | 0.039 | | 0.022 | 0.037 | |
| RCV1 | 0.079 | 0.097 | 0.091 | 0.105 | 0.088 | 0.103 | | 0.082 | 0.098 | |
| RotaC | 0.087 | 0.096 | 0.100 | 0.104 | 0.097 | 0.102 | | 0.089 | 0.098 | |
| YFV | 0.030 | 0.017 | - | - | 0.033 | 0.018 | | - | - | |
| JE | 0.101 | 0.063 | - | - | - | - | | 0.104 | 0.064 | |
| **Total** | **1.00** | **1.00** | **1.00** | **1.00** | **1.00** | **1.00** | | **1.00** | **1.00** | |

Supplementary Table 2: The difference in vaccine uptake (%) between 2006 and 2016

|  | **BCG** | **DTP3** | **HepB3** | **Hib3** | **IPV1** | **MCV2** | **PCV3** | **Pol3** | **RCV1** | **RotaC** | **YFV** | **JE** |
| --- | --- | --- | --- | --- | --- | --- | --- | --- | --- | --- | --- | --- |
| **Indonesia** | -7 | 7 | 13 | 79 | 2 | 6 |  | 2 |  |  |  |  |
| **Malaysia** | 0 | 3 | 3 | 9 | 91 | 9 |  | 3 | 1 |  |  | 98 |
| **Philippines** | -15 | -2 | 9 | 86 | 37 | 66 | 36 | -16 | 80 |  |  |  |
| **China** | 7 | 6 | 8 |  |  | 5 |  | 5 | 99 |  |  | 99 |
| **Thailand** | 0 | 0 | 3 |  |  | 1 |  | -1 | -3 |  |  | 5 |
| **Vietnam** | 0 | 2 | 3 | 96 |  | 95 |  | 1 | 99 |  |  | 0 |
| **Sri Lanka** | 0 | 1 | 1 | 99 | 99 | 1 |  | 1 | 1 |  |  | 29 |
| **Kazakhstan** | -4 | -17 | -17 | 82 | 93 | 0 | 97 | -17 | 0 |  |  |  |
| **Brazil** | 0 | -13 | -13 | -11 | 80 | 17 | 94 | -1 | -3 | 47 | 5 |  |
| **Colombia** | -8 | -2 | -2 | -2 | 92 | 2 | 89 | -3 | -2 | 90 | 4 |  |
| **Egypt** | -3 | -3 | -3 | 94 |  | -1 |  | -3 | -2 |  |  |  |
| **Morocco** | 4 | 2 | 4 | 99 | 95 | 99 | 98 | 2 | 7 | 99 |  |  |
| **Bulgaria** | -2 | -3 | -5 | 92 |  | -5 | 90 | -4 | -4 |  |  |  |
| **Romania** | -15 | -8 | -9 | 89 |  | -20 |  | -8 | -9 |  |  |  |
| **Jordan** | 4 | 0 | 0 | 0 | 99 | 11 |  | 0 | 11 | 97 |  |  |

Supplementary Figure 1: The change in per capita expenditure on vaccines across investigated countries.


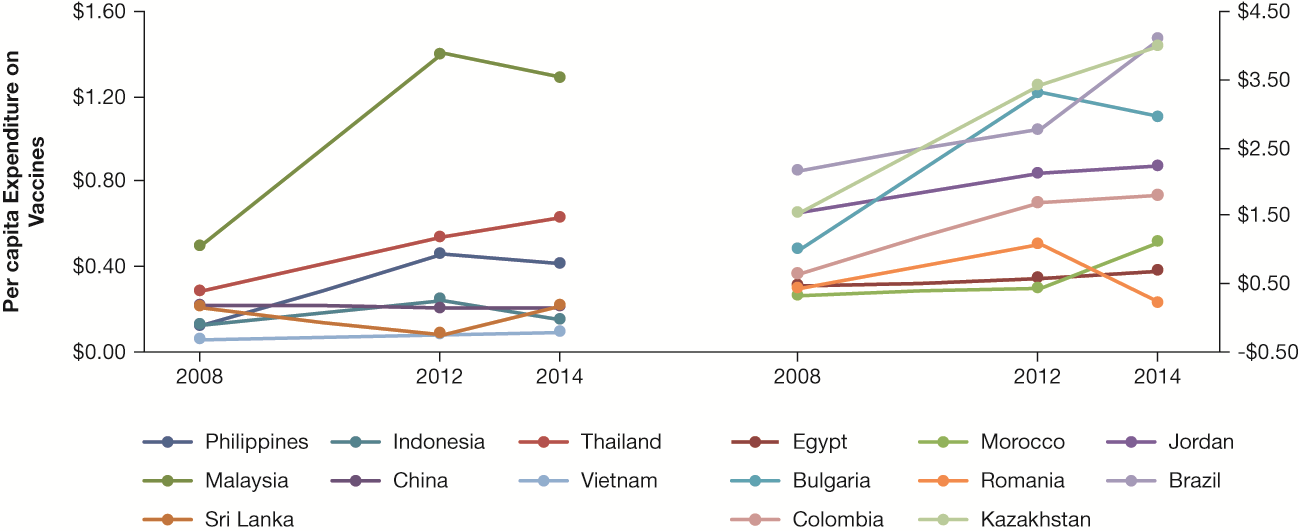


| Country | Vaccination Commitment (WAIVC)^[[1]](#footnote-1)^ | Under 5 Infant Mortality^[[2]](#footnote-2)^ | Life Expectancy^[[3]](#footnote-3)^ |
| --- | --- | --- | --- |
| **Asia-Pacific** | 0.4749 | -0.4948 | 0.5820 |
| Indonesia | 0.1958 | -0.2918 | 0.2706 |
| Malaysia | 0.4051 | 0.2071 | 0.7421 |
| Philippines | 0.8214 | -0.9303 | 0.9047 |
| China | 0.3146 | -0.5999 | 0.4299 |
| Thailand | 0.3790 | -0.6634 | 0.5764 |
| Vietnam | 0.9089 | -0.9161 | 0.9165 |
| Sri Lanka | 0.2995 | -0.2696 | 0.2336 |
| **Latin America** | 0.5277 | -0.6543 | 0.7297 |
| Brazil | 0.6871 | -0.8085 | 0.7499 |
| Colombia | 0.3684 | -0.5001 | 0.7095 |
| **Africa and Middle East** | 0.5356 | -0.6212 | 0.6381 |
| Egypt | 0.1042 | -0.1127 | 0.1736 |
| Morocco | 0.7135 | -0.8508 | 0.7997 |
| Jordan | 0.7892 | -0.9000 | 0.9410 |
| **Europe** | 0.5832 | -0.2302 | 0.5561 |
| Bulgaria | 0.8532 | -0.5697 | 0.7210 |
| Romania | 0.0327 | 0.1487 | 0.0160 |
| Kazakhstan | 0.8638 | -0.2696 | 0.9313 |
| **Total** | **0.5158** | **-0.4884** | **0.6077** |

Supplementary Table 3: Calculated regional and country specific correlations between per capita government expenditure and health outcomes

1. The data for total and per capita government expenditure on vaccines was obtained from WHO / UNICEF Joint Reporting Form: WHO Financing Immunisation Indicators [32] [↑](#footnote-ref-1)
2. The data for under-5 infant mortality was obtained from The World Bank Data Bank: Mortality rate, under-5 (per 1,000 live births) [16]. [↑](#footnote-ref-2)
3. The data for life expectancy at birth was obtained from The World Bank Data Bank: Life expectancy at birth, total (years) [33]. [↑](#footnote-ref-3)
